# Neighborhood environments and self-rated health in Mainland China, Japan and South Korea

**DOI:** 10.1371/journal.pone.0204910

**Published:** 2018-09-27

**Authors:** Jing Liu, Ye Luo, William Haller, Brenda Vander Mey, Ellen Granberg

**Affiliations:** 1 Department of Sociology, Georgia State University, Atlanta, Georgia, United States of America; 2 Department of Sociology, Anthropology and Criminal Justice, Clemson University, Clemson, South Carolina, United States of America; 3 Office of the Provost, Rochester Institute of Technology, Rochester, New York, United States of America; University of Texas Medical Branch at Galveston, UNITED STATES

## Abstract

Neighborhood environments are considered crucial determinants of self-rated health. Previous research has documented a positive association between the quality of neighborhood environments and health status. However, the relationship between neighborhood environments and health status in East Asian countries has received far less attention. This study examined the relationship between the three main types of neighborhood environments (built, natural, and social) and self-rated health in Mainland China, Japan, and South Korea. It also compared the neighborhood effects on self-rated health across the three countries. Our analytical sample included 3,675, 2,390, and 1,500 respondents in China, Japan, and South Korea respectively from the 2010 East Asian Social Survey. Ordinal Logistic Regression models were estimated for each country and the country differences were tested. This study found that neighborhood built, natural and social environments are positively associated with self-rated health in China, Japan and South Korea. These effects vary somewhat by country, and neighborhood social environment has the strongest association with self-rated health in Japan, followed by South Korea and then China. The similar relationship between perceived neighborhood environments and self-rated health across the three countries underscores the prevalent impact of perceived neighborhood environments on health. The greater association between social environment and self-rated health in Japan suggests the greater need of community based support system in an aging society not only for the older people, but also for the general population, especially those who are living in poor neighborhood social environment.

## Introduction

Neighborhood environments are considered crucial factors in affecting health. Previous empirical research has documented a positive association between self ratings of neighborhood environments and health status. This relationship has been extensively studied in western countries [1, 2, 3–5, 6]. Although similar studies related to neighborhood environments and self-rated health have focused on single countries in East Asia [7–9], comparison across countries has received far less attention. In the past few decades, many East Asian countries have experienced rapid industrialization which resulted in tremendous changes to people’s living environments. This rapid industrialization has also been associated with increased life expectancy and living standards [7, 10]. Previous research showed that, with higher life expectancy and living standards, people tend to care more about their health and spend more on their health care [11, 12]. This is especially true in Mainland China, Japan, and South Korea which lead this industrialization process in East Asia. However, little is known about how living environments may have affected people’s health and which environments play a more crucial role in affecting health in these countries.

This study examines the relationship between three types of neighborhood environments and self-rated health in East Asian countries. These three types are the built environment, the natural environment, and the social environment. The relationships between neighborhood environments and health are compared across China, Japan and South Korea. Findings from this study will contribute to the literature on the linkages between neighborhood environments and health. More importantly, studying the effects of neighborhood environments on self-rated health in a cross-national context will not only contribute to a better understanding of the various causes of inequality in health, but also aid policy makers in China, Japan and South Korea to make more effective policies to promote healthier living environments and mitigate possible adverse effects of neighborhood environments on their population’s health.

### The effects of neighborhood environments on health

Collective efficacy theory is a useful theoretical framework for studying the relationship between neighborhood environments and health [13–16]. Collective efficacy theory is rooted in the social disorganization perspective and shares its emphasis on structural disadvantage and the prevalence of social network at the community level, but the collective efficacy theory moves beyond a narrow focus on network ties to emphasize mutual trust and solidarity among local residents, and principally, expectations for action in explaining the impact of neighborhood factors on residents’ well-being [13]. According to this theory, neighborhood environments can affect health through their impact on collective efficacy which is defined as the process of activating or converting social ties among neighborhood residents in order to achieve collective goals, such as public order or the control of crime [17, 18]. There are several possible reasons that collective efficacy may affect health [13, 19]. First, risky and problem behaviors (such as substance abuse, child abuse and reckless behavior) may be subject to the efforts of collective social control [18]. Second, neighborhoods with higher levels of collective efficacy may be more effective at attracting and maintaining health and education services than those neighborhoods with lower collective efficacy. Third, many physical hazards in the community, such as decaying community infrastructure (poorly maintained streets) and housing stock (dilapidated or abandoned buildings) could have negative effects on the community. These risks could be more effectively reduced in the neighborhoods with high collective efficacy through the solicitation of external resources to correct potentially risky conditions and through rigorous monitoring of neighborhood hazards and vulnerable residents, such as the elderly or disabled [20, 21]. Finally, collective efficacy may improve health and well-being through psychosocial processes such as reducing fear and promoting self-respect [13].

Neighborhood environments can be generally categorized into three types, which are built, natural and social environments, and they can be strong determinants of people’s health status [5, 22–24]. Built environment refers to the man-made surroundings that provide the setting in which people live, work, and recreate on a day-to-day basis. It consists of neighborhood roads, buildings, food sources, and other recreational facilities. It affects many daily decisions, such as whether to walk to work or school, to do physical exercise, to eat frequently at fast food restaurants, or to take children to parks, all of which can have important health consequences [3, 15, 25–30]. Research has shown that people are usually physically and mentally healthier in neighborhoods with healthier food choices, and with more sports and recreational facilities [24, 31]. On the other hand, greater perceptions of built environment problems in neighborhoods result in a lower quality of life, deteriorating physical functioning and many specific disease outcomes [32]. While most studies focused on the West, there is some evidence of a positive association between the quality of built environment and health outcomes in China [33], Japan [34], and South Korea [35].

Natural environment refers to all living and non-living things occurring naturally. It encompasses the interaction of all living species, climate, weather, and natural resources that affect human survival and economic activity [36]. This study focuses particularly on the air, water and noise pollution of neighborhoods as natural environment indicators. Ellen et al. indicated that the neighborhood natural environment affects self-rated health through polluting factories and toxic waste sites [1]. There are also studies related to pollution and health in East Asia. For example, air pollutants, such as automobile exhaust, are shown to be a major cause of asthma in Japan [37, 38]. Wen and Gu found that air pollution can shorten population’s life expectancy and health expectancy for older adults in China [39]. Air pollution was also linked to multiple adverse health outcomes in South Korea [40].

Social environment refers to the immediate physical surroundings and social relationships, as well as cultural milieus, within which defined groups of people live and interact. It includes social networks, neighborhood safety and social support [41]. A large body of research has shown that people who have more extensive and strong social connections and social support report better health and lower mortality risk [20, 41, 42]. Wen et al. found that neighborhood satisfaction, social cohesion and safety are strongly associated with self-rated health in China [9]. Lee et al. showed that trust of neighbors and exchanging help with neighbors are positively associated with self-rated health in South Korea [35]. Several studies showed that neighborhood social environment is associated with better physical health in Japan [43–45]. Hence our first hypothesis is:

*H1*: *The quality of neighborhood built*, *natural*, *and social environments are positively associated with self-rated health status in China*, *Japan and South Korea*.

### Neighborhood effects on health in the East Asian context

Mainland China, Japan and South Korea are three leading economies in East Asia. The three countries share a deep-rooted Confucian cultural tradition which values collectivism, harmony and mutual respect. They differ, however, in many other aspects, and thus some differences can be expected in the neighborhood environments and health relationships among these countries. In the past few decades, as a developing country, China has achieved rapid industrialization. At the same time, life expectancy and living standards have also increased. More people are more concerned about the quality of what they eat and where they live [46]. Therefore, the built environment in China becomes more crucial when its population considers their health status. As developed countries affected by western cultures for decades, Japan and South Korea have a relatively long history of economic development and raising their populations’ living standards [47, 48]. The built environment in these countries are characterized by well-developed commercial areas with good access to public transit [30]. High population density, however, remains a major concern in these countries. Although previous research found an association between built environment and health in each country, it is not clear whether the strength of this association varies across these countries.

As a result of the economic growth, China is faced with an unprecedented environmental threat as a trade-off for its developing economy, especially air and water pollution, which have become two major sources of morbidity and mortality in China. As China increasingly contributes to global economic growth, the country also potentially becomes one of the largest polluters in the world [49], which raises environmental concern among Chinese citizens, especially serious among those who are more educated, males, residents of large Chinese cities, and government employees [50]. Serious air pollution increases the risk of lung cancer among Chinese population. Previous research showed that lung cancer is a serious health problem in China, as in the rest of the world [49]. Tie and colleagues also found that air pollution poses a considerable risk for respiratory morbidity, cardio-pulmonary mortality and the incident of lung cancer [51]. This particular situation may amplify the natural environment’s effects on the Chinese population’s health status. Since Japan and South Korea have different geographic, political, and historical contexts than China, the situation may vary for these two countries. Japan and South Korea have a relatively longer history of environmental pollution control as both countries started industrialization earlier than China. Environmental pollution became an issue in the 19th century in Japan. In 1967, Japan enacted the Basic Law for Environmental Pollution Control and since then, Japan has made several public efforts to reduce environmental pollution [52]. In South Korea, air quality in the Seoul metropolitan area deteriorated significantly during the country’s rapid industrialization in the 1970s and the government has made significant success in improving water and air quality and conserving ecosystems by introducing various environmental regulations [53]. However, natural disasters and industrial accidents as illustrated by the Fukushima Daiichi disaster of 2011 in Japan complicate the situation more than previously supposed. These damaging factors affect population’s health status both physically and psychologically. A recent study showed that physical health is associated with air and noise pollution in Japan, but not in China and South Korea which the authors attributed to the higher expectations of Japanese citizens for a clean environment [54]. Based on the limited evidence, we tentatively expect that natural environment has greater impact on self-rated health in Japan than in China and Korea.

The association between neighborhood social environment and self-rated health may also vary by country and there are several reasons that neighborhood social environment may matter the most for Japan. First, according to the United Nations’ report, although all three countries are facing rapid population aging due to declining fertility rate and increasing life expectancy, Japan is one of the foremost aged societies in the world with 26 percent of its population being aged 65 and older in 2015, while the proportion of this age group was 13 percent in South Korea and 9.7 percent in China in the same year [55]. At the same time, Japan has the lowest proportion of older adults living with children [56]. Previous research showed that the effect of neighborhood social environment on health is particularly important for older adults because of their longer duration of exposure, increased biological and psychological vulnerability, changing patterns of special usage with age, and a greater reliance on access to community sources of integration when other social networks contract [23, 57–59]. Second, the proportion of never married individuals is higher in Japan than in China. A study based on 2006 East Asian Social Survey showed that the proportion of the never married between ages 20 and 79 in Japan was nearly three times as large as in China (21.3 percent versus 7.3 percent) [60]. For singles, social support from neighbors are likely to be more important because they do not receive support from spouse as married individuals do. Third, although China has experienced rapid urbanization since the onset of its economic reform in the 1970s, China is still far behind than Japan and South Korea in the level of urbanization. According to the United Nations’ estimates, 57 percent of China’s population lived in urban areas in 2016, while 94 percent of Japanese and 83 percent of South Koreans lived in urban areas [61]. In China, there is a large urban-rural divide in economic and welfare resources. With a large number of working aged adults moving to the cities, there is a dire need of social support from communities for rural residents. However, previous research showed that rural residents in China had higher mortality rate and higher prevalence of functional limitations which was in part due to worse and limited supportive resources and infrastructure to facilitate social connectedness when the working aged adults moved to the city [33]. Urban residents, on the other hand, may rely more on neighbors because of smaller family size.

Based on these country differences, we hypothesize that:

*H2*: *The relationships between neighborhood environments and self-rated health vary among China*, *Japan and South Korea*. *More specifically*, *the natural and social environments have stronger effects on self-rated health status in Japan than in China and South Korea*.

## Methods

### Data

Data for this study come from the 2010 East Asian Social Survey (EASS) Data Archive. The EASS is a biennial social survey project that serves as a cross-national network of GSS-type surveys in East Asian (http://www.eassda.org). The surveys included in the EASS has a common module, like the US General Social Survey, and it was designed almost uniformly for all countries which make them largely comparable with each other. Our study was based on data from the Chinese, Japanese, and South Korean General Social Surveys (CGSS 2010, JGSS 2010 and KGSS 2010, respectively). Multi-stage stratified random sampling in each country produced nationally representative samples of 3,866 respondents in China with a response rate of 72%, 2,496 respondents in Japan with a response rate of 56%, and 1,576 respondents in South Korea with a response rate of 63%. Data were collected through in-person interviews in China and South Korea and through both in-person interviews and self-administered surveys in Japan. The original collector of the data obtained ethical approval and made the data public available for secondary data analysis. Because the target populations of the original surveys were adults aged 18 years and older for the CGSS and KGSS and adults aged between 20 and 89 years for the JGSS, we restricted our analysis to the 7,817 respondents aged between 20 and 89 years. Because the number of missing cases for most variables used in this study was very small, we deleted missing cases listwise except for household income for which multiple imputation was used to impute values for the missing cases. Our analytical sample included 3,675, 2,390, and 1,500 respondents in China, Japan, and South Korea respectively.

### Measures

*Self-rated Health*.—Respondents were asked to rate their health on a five-point scale (1 = Poor, 2 = Fair, 3 = Good, 4 = Very good, 5 = Excellent). Self-rated health is a commonly used measure of overall health. According to Idler and Benyamini [62], self-rated health can capture the full array of disease that people have, and sometimes even symptoms relating to an undiagnosed illness. Also, self-rated health reflects the impact of collective efficacy more readily than any other measures of health status due to its nature and scope of the question [63].

*Neighborhood Environments*.*—*The neighborhood environments were measured by respondents’ own reports. Many previous studies have found that both objective neighborhood characteristics [64–66] and subjective neighborhood characteristics [67, 68] were related to health. However, the subjective aspect was not only strongly associated with health, but also could mediate associations between the objective aspects (neighborhood disadvantage and affluence) and health when being examined at the same time [69, 70]. Three dimensions of neighborhood environments were assessed. (1) *Perceived neighborhood built environment*. The respondents were first informed that “neighborhood” is defined as the area 1 kilometer (approximately 15 minutes on foot) around respondent’s residential area and then were asked to what extent they agree or disagree with the following three statements: (i) “The neighborhood is suitable for doing exercise such as jogging or walking”; (ii) “A large selection of fresh fruits and/or vegetable is available in the neighborhood”; and (iii) “The neighborhood has adequate public facilities such as community centers, library, parks, etc.” The five response categories for each question range from 1 = Strongly disagree to 5 = Strongly agree. (2) *Perceived neighborhood natural environment*. This was derived from three questions asking the respondents how severe (i) the air, (ii) water, and (iii) noise pollution are in the area of their local residence. The four response categories for each question range from 1 = Very severe to 4 = Not severe at all. (3) *Perceived neighborhood social environment*. The respondents were also first informed that “neighborhood” is defined as the area 1 kilometer (approximately 15 minutes on foot) around respondent’s residential area and then were asked to what extent they agree or disagree with the following three statements: (i) “The neighbors are mutually concerned for each other”, (ii) “The neighborhood is safe”, and (iii) “Neighbors are willing to provide assistance when I am in need”. The five response categories range from 1 = Strongly disagree to 5 = Strongly agree. Factor analysis clearly identified these three factors among the nine items with each factor accounting for 21–26% of the total variance. Cronbach’s alpha is .632 for the build environment index, .823 for the natural environment index and .756 for the social environment index.

We created three neighborhood environment indexes by averaging responses to the three indicators of a particular dimension of neighborhood environment. The correlation was .06 between built and natural environments, .15 between built and social environments, and .27 between national and social environments, suggesting these are three distinct albeit related aspects of neighborhood environments. In addition, previous research showed that the Japanese are less likely to express pros and cons clearly and their answers to agreement scales tend to concentrate on a mid-point [71], and thus a one-point increase in a particular index in one country may have a different meaning and cannot be directly compared to one in another. To adjust for this tendency, we also created standardized neighborhood environment indexes by standardizing responses to each question within each country and then averaging the standardized scores of the three items in each neighborhood environment index. We report descriptive statistics for the unstandardized indexes and report regression results using standardized indexes.

*Control Variables*.*—*The control variables include sociodemographic characteristics that are likely to affect both perceptions of neighborhood environments and self-rated health, including respondent’s age in years, gender, marital status (married, widowed, divorced/separated, and never married), years of schooling, household income, employment status and urban/rural residence. The household income variables were originally ratio variables in the CGSS and KGSS, and an ordinal variable in the JGSS, and were included in the dataset as separate variables for each country. Because household income was missing for 13% of Chinese respondents, 27% of Japanese respondents, and 12% of South Korean respondents, we imputed missing cases based on sociodemographic variables, the “top-bottom self-placement” question which asked respondents to place themselves on a 10-point scale in terms of their socioeconomic standing, and a question asking respondents to compare their family’s economic status to other families in the same area on a 5-point scale ranging from “far below average” to “far above average.” Considering the socioeconomic and currency differences in the three countries, household income for each country was recoded into four categories roughly representing the four quartiles with household income below 25% as the reference category.

### Statistical analysis

We weighted our analysis using the weight provided in the original surveys so that the findings can be generalized to all adults aged 20 to 89 years in each country. Descriptive statistics stratified by country were calculated for all variables. The Kruskal-Wallis test was used for testing differences in self-rated health status, built, natural, and social environments, age, and years of schooling among the three countries because this test is not subject to normal distribution and equal variances assumptions. The Chi-square test was used for testing differences in gender, urban/rural residence, marital status, employment status, and household income among these countries.

Four ordinal logistic regression models were estimated for each country, which examine whether neighborhood built, natural and social environments could separately or jointly affect self-rated health status. The proportional odds assumption for ordinal regression was met by most models. Additional analyses using ordinal least squares regression produced similar results. In models 1, 2, and 3, each of the three standardized environment indexes was entered separately in the regression model for each country. In model 4, the three environment indexes were added into the model together to examine whether each of the three neighborhood environments is still significant in predicting self-rated health, when controlling for other two types of neighborhood environments. Respondent’s age, gender, marital status, years of schooling, household income, employment status and urban/rural residence were controlled for in all four models. *SUEST* and *TEST* procedures in Stata were used to test whether the coefficients are significantly different among these countries.

## Results

### Descriptive statistics

Table 1 shows the descriptive statistics for each variable in this study. Overall, a large majority of respondents reported good, very good or excellent health statuses. In China, a higher proportion of respondents rated their health status as very good, which accounts for 34.5% of all respondents in this study; 3.7% respondents reported their health status as poor, 13.6% reported fair health, 23.3% reported their health status as good, and 25% rated their health as excellent. In South Korea, a higher proportion of respondents also rated their health status as very good (about 30.3%); 9.1% reported poor health, 14.8% reported fair health, 24.9% rated their health status as good, and 20.9% rated their health as excellent. In Japan, most respondents rated their health statuses as good (about 51.7%); 3.7% respondents reported poor health status, 24.5% reported fair health, 16.9% reported very good health, and 3.2% reported excellent health.

**Table 1:** Descriptive statistics for respondents in China, Japan, South Korea.

Variables	China (*N* = 3675)	Japan (*N* = 2390)	South Korea (*N* = 1500)	*P* of country differences
	Mean (STD) / %	Mean (STD) / %	Mean (STD) / %	
Self-Rated health status				**
Poor	3.7%	3.7%	9.1%	
Fair	13.6%	24.5%	14.8%	
Good	23.3%	51.7%	24.9%	
Very good	34.5%	16.9%	30.3%	
Excellent	25.0%	3.2%	20.9%	
Built environment index	3.12 (.89)	3.80 (.76)	3.66 (.99)	**
Natural environment index	2.94 (.75)	3.14 (.65)	2.65 (.69)	**
Social environment index	3.91 (.73)	3.56 (.77)	3.21 (.95)	**
Age	45.49 (14.84)	51.64 (18.06)	45.77 (16.02)	**
Sex				n.s.
Male	48.5%	48.8%	47.3%	
Female	51.5%	51.2%	52.7%	
Residence				**
Urban	58.7%	64.9%	86.3%	
Rural	41.3%	35.1%	13.7%	
Marital status				**
Married	85.1%	68.5%	66.1%	
Widowed	5.5%	8.4%	7.9%	
Divorced	1.8%	3.7%	4.7%	
Never married	7.6%	19.4%	21.3%	
Years of schooling	8.30 (4.37)	12.72 (2.52)	11.95 (4.32)	**
Household income				**
Low	22.6%	15.9%	24.7%	
Relatively low	25.7%	26.7%	20.1%	
Relatively high	24.8%	32.2%	30.0%	
High	26.9%	25.3%	25.1%	
Work status				**
Working	67.7%	62.1%	61.0%	
Not working	32.3%	37.9%	39.0%	

Note: Results based on 2010 EASS. The numbers are weighted. The Kruskal-Wallis test was used to test country differences for self-rated health status, built, natural and social environments, age and years of schooling. The Chi-square test was used for gender, residence, marital status, household income and work status.

** *p* < .01.

For the built environment index and on a scale from 1 to 5, Japan has the highest mean score among the three countries (M = 3.80, SD = .76), followed by South Korea (M = 3.66, SD = .99) and China (M = 3.12, SD = .89). For the natural environment index and on a scale from 1 to 4, Japan has the highest mean score (M = 3.14, SD = .65), followed by China (M = 2.94, SD = .75) and South Korea (M = 2.65, SD = .69). For the social environment index and on a scale from 1 to 5, China has the highest average score (M = 3.91, SD = .73), followed by Japan (M = 3.56, SD = .77) and South Korea (M = 3.21, SD = .95).

For the control variables, the results show that a majority of respondents are from urban areas. The average age is highest in Japan (M = 51.64, SD = 18.06), and it is similar for South Korea (M = 45.77, SD = 16.02) and China (M = 45.49, SD = 14.84). For Chinese respondents, 85.1% were married, 5.5% were widowed, 1.8% were divorced, and 7.6% were never married. In Japan, 68.5% respondents were married, 8.4% were widowed, 3.7% were divorced, and 19.4% were never married. In South Korea, 66.1% respondents were married, 7.9% were widowed, 4.7% were divorced, and 21.3% were never married. Education ranged from 0 to 21 years, and the average years of education was highest in Japan (M = 12.72, SD = 2.52) and lowest in China (M = 8.30, SD = 4.37). Because household income was recoded into quartiles separately for each country, the percentage distribution should be similar for the three countries. However, because the cut-points used were not precise, we see some differences in the percentage distributions. China had the highest proportion of respondents who were employed (67.7%) and a similar proportion of respondents were employed in Japan (62.1%) and South Korea (61%). In this study, a higher proportion of respondents were females and this was the only variable which did not significantly differ among the three countries.

### Effects of neighborhood environments on self-rated health in China, Japan and South Korea

Table 2 shows results from the four ordinal logistic regression models of neighborhood environment effects on self-rated health. In Model 1, only the effect of built environment on self-rated health was tested. After controlling for sociodemographic covariates, built environment shows a significant positive association with self-rated health in all three countries. This association is the strongest in Japan; one point increase in the standardized built environment index is associated with 30% increase in the likelihood of reporting better health in Japan while the increase is 23% in China and 18% in South Korea. The difference in the association between built environment and self-rated health is not statistically significant among the three countries.

**Table 2:** Ordinal regression models of the relationships between neighborhood environments and self-rated health for adults in China, Japan and South Korea.

Variables	Model 1			Model 2			Model 3			Model 4		
	China	Japan	South Korea	China	Japan	South Korea	China	Japan	South Korea	China	Japan	South Korea
Built environment (standardized)	1.23** (.06)	1.30** (.07)	1.18** (.07)							1.18** (.05)	1.07 (.06)	1.07 (.07)
Natural environment (standardized)				1.11** (.04)	1.23** (.06)	1.21** (.07)				1.06 (.04)	1.15** (.05)	1.15* (.07)
Social environment (standardized)							1.22** (.05)	1.49**^a^ (.08)	1.35** (.09)	1.17** (.05)	1.40**^a^ (.08)	1.27** (.09)
Age	.96** (.003)	.97**^a^ (.003)	.97** (.005)	.96** (.003)	.97**^a^ (.003)	.97** (.005)	.96** (.003)	.97** (.003)	.96** (.005)	.96** (.003)	.96** (.003)	.96** (.005)
Male	1.30** (.08)	1.07^a^ (.09)	1.42**^c^ (.14)	1.31** (.08)	1.03^a^ (.08)	1.42**^c^ (.14)	1.32** (.08)	1.07^a^ (.09)	1.46**^c^ (.15)	1.31** (.08)	1.05^a^ (.09)	1.46** ^c^ (.15)
Urban residence	1.00 (.07)	1.02 (.09)	1.13 (.17)	1.13 (.08)	1.08 (.09)	1.24 (.19)	1.14* (.08)	1.00 (.09)	1.29* (.20)	1.11 (.09)	1.13 (.10)	1.28 (.20)
Marital status (ref = Married)												
Widowed	.89 (.13)	1.40*^a^ (.22)	1.04 (.23)	.88 (.13)	1.30^a^ (.21)	1.00 (.22)	.90 (.13)	1.34*^a^ (.21)	1.05 (.23)	.90 (.13)	1.31* (.21)	1.04 (.23)
Divorced	.69* (.15)	.69* (.15)	1.06 (.24)	.69* (.15)	.66** (.15)	1.01 (.23)	.73 (.16)	.70 (.16)	1.10 (.25)	.72 (.16)	.69* (.15)	1.08 (.24)
Never married	.94 (.12)	.74** (.09)	.98 (.14)	.93 (.12)	.71** (.08)	.97 (.14)	.96 (.12)	.73** (.09)	1.00 (.15)	.96 (.12)	.72** (.08)	.99 (.14)
Years of schooling	1.02** (.01)	1.02 (.02)	1.08**^b^^c^ (.02)	1.03** (.01)	1.03 (.02)	1.08**^b^^c^ (.02)	1.03** (.01)	1.03 (.02)	1.08**^b^^c^ (.02)	1.03* (.01)	1.03 (.02)	1.08**^b^^c^ (.02)
Household income (ref = Low)												
Relatively low	1.44** (.13)	1.37* (.18)	1.51** (.24)	1.44** (.13)	1.36* (.18)	1.52** (.24)	1.42** (.13)	1.35* (.18)	1.53** (.25)	1.43** (.13)	1.33* (.17)	1.53** (.24)
Relatively high	1.77** (.17)	1.57** (.21)	1.41* (.22)	1.80** (.17)	1.56** (.21)	1.45* (.22)	1.77** (.17)	1.48** (.20)	1.42* (.22)	1.77** (.17)	1.46** (.20)	1.41* (.22)
High	2.00** (.20)	1.96** (.29)	1.80** (.30)	2.07** (.21)	1.97** (.29)	1.82** (.31)	2.08** (.20)	1.81** (.27)	1.78** (.30)	2.03** (.20)	1.80** (.27)	1.73** (.29)
Working	1.38** (.10)	1.11 (.11)	1.30* (.14)	1.34** (.10)	1.13 (.11)	1.30* (.14)	1.35** (.10)	1.16 (.11)	1.32** (.14)	1.36** (.10)	1.17 (.12)	1.34** (.14)
*X*^*2*^	755.42	286.52	390.94	741.68	282.54	394.59	759.68	325.15	405.51	775.65	335.25	411.80
*Df*	12	12	12	12	12	12	12	12	12	14	14	14
*N*	3,675	2,390	1,500	3,675	2,390	1,500	3,675	2,390	1,500	3,675	2,390	1,500

Note: Numbers are odds ratios with standard errors in parentheses. The results are weighted.

*p < .05

**p < .01.

^a^ indicates the odds ratio is significantly different between China and Japan at *p* < .05 level.

^b^ indicates the odds ratio is significantly different between China and South Korea at *p* < .05 level.

^c^ indicates the odds ratio is significantly different between Japan and South Korea at *p* < .05 level.

Model 2 examines whether the neighborhood natural environment has any effect on self-rated health when only sociodemographic covariates are controlled for. Neighborhood natural environment is a significant positive predictor for self-rated health in all three countries. This association is the similar in Japan and South Korea. In Japan, one point increase in the standardized natural environment index is associated with 23% increase in the likelihood of reporting better health and this increase is 21% in South Korea, while this increase is only 11% in China. The difference in the association between natural environment and self-rated health is not statistically significant among the three countries.

Model 3 examines whether the social environment has any effect on self-rated health after controlling for sociodemographic covariates. The social environment shows a significant positive association with self-rated health in all three countries. Once again, this association is the strongest in Japan with one point increase in the standardized social environment index associated with 49% increase in the likelihood of reporting better health. This increase is 35% in South Korea and 22% in China. The difference between China and Japan is statistically significant.

Model 4 includes all three neighborhood environment measures and sociodemographic covariates. After controlling for natural and social environments, the association between built environment and self-rated health is only significant in China. Neighborhood built environment is still positively associated with self-rated health in China. However, this effect is attenuated when the other two types of neighborhood environments are added in the model. When built and social environments are controlled for, the association between natural environment and self-rated health is no longer statistically significant in China. This association is also attenuated for Japan and South Korea, but remains significant. The association between neighborhood social environment and self-rated health is also attenuated for all three countries after controlling for natural and built environments, but neighborhood social environment is still significant for predicting self-rated health in all three countries. Also, this association is significantly stronger in Japan than in China.

Besides neighborhood environments, some sociodemographic variables are also significant predictors of self-rated health. For example, age has a negative association with self-rated health, while household income is positively associated with self-rated health in all three countries. Gender, education and work status are significant predictors in China and South Korea with males, more educated and those who are employed reporting better health, but these three variables do not have a significant independent association with self-rated health in Japan. Marital status is a strong predictor for self-rated health in Japan with those who were divorced/separated and never married reporting poorer health than those who were currently married. The effects of these sociodemographic variables vary somewhat in the four models but they do not change substantially.

Because self-rated health is strongly associated with chronic illness and there is some evidence that neighborhood environments are associated with the onset of chronic diseases [72], the observed associations between neighborhood environments and self-rated health in Table 2 may be explained by the effects of neighborhood environments on chronic conditions. On the other hand, people suffering from chronic illness may have different preferences for and perceptions of their environments because of their greater reliance on them. Although with the EASS data we cannot determine whether chronic illness is a mediator or a confounder, we conducted a sensitivity test in which we reran the same models as those in Table 2, but added a dummy variable for chronic illness (coded 1 if the respondent had any chronic diseases or longstanding health problems) as a covariate and the results are presented in Table 3. With chronic illness controlled for, the coefficients of neighborhood environment variables changed somewhat, but they all remain significant when added separately. One notable change is that the association between built environment and self-rated health in Japan is larger when chronic illness is controlled for and it remains significant when all environment variables are included in model 4. This is mainly due to a positive association between chronic illness and neighborhood built environment in Japan.

**Table 3:** Ordinal regression models of the relationships between neighborhood environments and self-rated health for adults in China, Japan and South Korea including chronic illness as a covariate.

Variables	Model 1			Model 2			Model 3			Model 4		
	China	Japan	South Korea	China	Japan	South Korea	China	Japan	South Korea	China	Japan	South Korea
Built environment (standardized)	1.22** (.06)	1.44**^a^ (.08)	1.22**^c^ (.06)							1.16** (.05)	1.21** (.07)	1.13* (.07)
Natural environment (standardized)				1.10* (.04)	1.17** (.05)	1.17** (.07)				1.04 (.04)	1.08 (.05)	1.11* (.07)
Social environment (standardized)							1.25** (.05)	1.52**^a^ (.08)	1.31** (.09)	1.21** (.05)	1.37** (.08)	1.22** (.08)

Note: Numbers are odds ratios with standard errors in parentheses. In addition to chronic illness, age, gender, residence, marital status, education, income, and work status are also controlled for in all models, but not shown in the table. The results are weighted.

*p < .05

**p < .01.

^a^ indicates the odds ratio is significantly different between China and Japan at *p* < .05 level.

^c^ indicates the odds ratio is significantly different between Japan and South Korea at *p* < .05 level.

## Discussion and conclusion

This study sought to fill the gap in the literature on the relationship between neighborhood environments and health by assessing the effects of neighborhood environments on self-rated health in China, Japan and South Korea. It also compared the relative importance of three types of neighborhood environments in the three countries. The results showed positive associations between neighborhood built, natural, and social environments and self-rated health in China, Japan and South Korea, which supports our first hypothesis. Respondents whose neighborhoods are suitable for exercise, have healthier food choices and more public facilities tend to report better health status than others. Respondents whose neighborhood are less polluted tend to report better health status. Respondents whose neighborhoods are safer and whose neighbors are more willing to help each other tend to report better health status. These findings are consistent with a large number of previous studies which suggest that neighborhood contexts are related to health independently of individual level attributes, and social influences on health operate in part through the types of neighborhoods in which people live in [1, 65, 68]. In addition, the finding that neighborhood environments are associated with self-rated health in all three countries extends previous research and suggests that neighborhood contexts may have impact on health in societies with different levels of economic development and different cultural contexts. Furthermore, the finding that all three types of neighborhood environments are associated with self-rated health demonstrates the importance of multiple dimensions of neighborhood environments for people’s health and well-being.

Our findings also provide new evidence for both social disorganization theory and collective efficacy theory. Our results showed that in these three East Asian countries, both neighborhood structural disadvantages, such as pollutions and the lack of community resources, and collective efficacy, such as trust, mutual concerns and mutual assistance, are associated with self-rated health even after their associations with individual level sociodemographic factors are taken into account. These findings are contrary to some studies in the West which found that neighborhood socioeconomic disadvantage is not significantly associated with self-rated health once individual level characteristics are controlled for [13]. It should be noted that our study and those studies on the West used different measures of neighborhood structural disadvantages and thus whether our findings reflect the unique social and cultural context in East Asia or they are the results of different measures used needs to be further investigated.

In this study, we found a stronger association between social environment and self-rated health in Japan than in China and South Korea even though only the difference between Japan and China is statistically significant. This finding partially supports our second hypothesis. Previous research suggests that since older people usually rely more on social support from neighbors, neighborhood social environment has stronger effects on self-rated health in aging countries [23, 73]. It should be noted that since age is controlled in our regression analysis, the observed differences in the association between neighborhood social environment and health among these countries cannot be solely attributed to the stronger impact of neighborhood social environment on older people’ health. Perhaps in an aging society, there is a greater need for high quality social environment not only for older people, but also for those who provide support to them. In addition, the difference in the proportion of the never married and different levels of urbanization in these countries may also partially explain the difference as singles and urban residents may rely more on neighbors for social support. Future research that look into the needs of social support of different sociodemographic groups in these countries would shed more light on the country differences we see here.

When adding the three types of neighborhood environments (built, natural and social) in one model (Model 4), the associations between each type of environment and self-rated health is substantially attenuated and some of them are no longer statistically significant. This suggests that certain types of environment are more salient in their impact on self-rated health. In China, built environment and social environment remain significant. For Japan and South Korea, natural environment and social environment remain significant (Table 2), but built environment becomes significant once chronic illness is controlled for (Table 3). One possible explanation that built environment is more strongly associated with self-rated health after controlling for chronic illness in Japan and South Korea is that people with chronic illness may have moved to neighborhoods which offer better services and more convenient living conditions, and in more developed countries like Japan and South Korea, people are more able to afford such moves. It is also interesting to note that the effect of social environment remains significant in all three countries even when other types of environments are controlled for, which suggests a more direct and stronger impact of social environment on health.

According to the results, perceived natural environment has the weakest association with self-rated health in China. This seems contradictory to what we would expect from these countries based on the different levels of pollution in the three countries. As a trade-off for rapid economic development, China is faced with an unprecedented environmental threat. However, Chinese people do not seem to be fully aware of the environmental threat they are facing. Our results show that Chinese respondents perceive better natural environment than South Korean respondents even though objective measures of environmental pollution indicate that China is more polluted than South Korea [74]. Thus, the weaker association between perceived natural environment and self-rated health in China should not be seen as evidence that natural environment has no impact on people’s health in China. In fact, this lack of awareness could have important implications for public health as people may be unwilling to take actions to avoid polluted air and water and to protect the environment.

Several limitations of this study should be noted. First of all, this study focused on self-rated health which does not necessarily represent all dimensions of health status. In addition, self-rated health is largely determined by the self-perception of various components of health and the weighted judgment of these components [75], and the criteria of self-rated health across the three countries may be different. Future research should look into other health measures, such as functional limitations and specific diseases. Second, the cross-sectional design of this study does not allow for the establishment of causality of the associations between neighborhood environments and self-rated health. Third, variables used to construct the three neighborhood environment indexes may only capture part of each of these environment dimensions. Four, due to data limitation, this study only examined the effects of perceived neighborhood environments. Although perceived environments are likely to have direct impacts on health, that of course does not preclude environmental effects that impact health before they become noticeable to those who are affected (e.g., lead in drinking water or carbon monoxide in homes). Future research that examines both objective and subjective environments may shed more light on the relationship between neighborhood environments and health. Fifth, for any type of neighborhood environment to take effect on health, one must be exposed to that environment for a sufficiently long time. This issue may affect young adults the most because they are more likely to move around. In additional analysis, we excluded respondents who were younger than 30 years old and found similar results. Still, future research needs to more directly examine the effect of duration of neighborhood residence on the relationship between neighborhood environments and health.

Despite these limitations, this study adds to a growing literature on the relationship between neighborhood environments and health in cross-national contexts. Findings from this study provide valuable information to the general public about the causes of social inequality in health which are not limited to individual sociodemographic characteristics. While social status matters to health, where you live and how you perceive it also matter. Findings from this study can also be informative to policymakers. The similar relationship between perceived neighborhood environments and self-rated health across the three countries underscores the prevalent impact of perceived neighborhood environments on health. To decrease the disparities in health, governments should pay attention not only to the objective measures of neighborhood environments, but also to how neighborhood environments are perceived by the residents as these two are not always parallel to each other and could have different health consequences. The variations in the effects of perceived neighborhood built, natural and social environments on self-rated health can aid policymakers in identifying priorities and making more effective policies to reduce possible adverse effects of poor neighborhood environments on people’s health. In addition, the greater association between social environment and self-rated health in a “super-aging society” [76] like Japan signifies the greater need of community based support system not only for the older people, but also for the general population, especially those who are living in poor social environment.
